# Inducing articular cartilage phenotype in costochondral cells

**DOI:** 10.1186/ar4409

**Published:** 2013-12-12

**Authors:** Meghan K Murphy, Grayson D DuRaine, A Hari Reddi, Jerry C Hu, Kyriacos A Athanasiou

**Affiliations:** 1 Department of Biomedical Engineering, University of California Davis, 451 E. Health Sciences Drive, Rm 2303, Davis, CA 95616, USA; 2 Department of Orthopaedic Surgery, University of California Davis, 4635 Second Avenue, Rm 2000, Sacramento, CA 95817, USA

## Abstract

**Introduction:**

Costochondral cells may be isolated with minimal donor site morbidity and are unaffected by pathologies of the diarthrodial joints. Identification of optimal exogenous stimuli will allow abundant and robust hyaline articular cartilage to be formed from this cell source.

**Methods:**

In a three factor, two level full factorial design, the effects of hydrostatic pressure (HP), transforming growth factor β1 (TGF-β1), and chondroitinase ABC (C-ABC), and all resulting combinations, were assessed in third passage expanded, redifferentiated costochondral cells. After 4 wks, the new cartilage was assessed for matrix content, superficial zone protein (SZP), and mechanical properties.

**Results:**

Hyaline articular cartilage was generated, demonstrating the presence of type II collagen and SZP, and the absence of type I collagen. TGF-β1 upregulated collagen synthesis by 175% and glycosaminoglycan synthesis by 75%, resulting in a nearly 200% increase in tensile and compressive moduli. C-ABC significantly increased collagen content, and fibril density and diameter, leading to a 125% increase in tensile modulus. Hydrostatic pressure increased fibril diameter by 30% and tensile modulus by 45%. Combining TGF-β1 with C-ABC synergistically increased collagen content by 300% and tensile strength by 320%, over control. No significant differences were observed between C-ABC/TGF-β1 dual treatment and HP/C-ABC/TGF-β1.

**Conclusions:**

Employing biochemical, biophysical, and mechanical stimuli generated robust hyaline articular cartilage with a tensile modulus of 2 MPa and a compressive instantaneous modulus of 650 kPa. Using expanded, redifferentiated costochondral cells in the self-assembling process allows for recapitulation of robust mechanical properties, and induced SZP expression, key characteristics of functional articular cartilage.

## Introduction

The poor innate healing capacity of articular cartilage often results in pain and loss of function. Cartilage lesions may originate from disease processes, from various genetic and metabolic conditions, or may be traumatically induced [1]. Whether originating from a disease process or trauma, articular cartilage lesions generally do not heal, or only partially heal resulting in inferior fibrocartilage [1]. Engineered articular cartilage may have the potential to replace degenerated tissues. However, the clinical success of tissue engineering relies on the development of mechanically and biochemically robust tissues, capable of withstanding *in vivo* loads upon implantation. Additionally, success relies on utilizing a cell source that is unaffected by pathology and is feasible for surgeons to isolate. Tissue engineering therefore presents a therapeutic approach that may address cartilage lesions, with the objective of reducing pain, restoring function, and halting joint degeneration.

Costal chondrocytes provide a clinically relevant cell source that may be suitable for autologous tissue engineering utilizing the self-assembling process [2,3]. Costal cartilage is located at the anterior ends of the ribs. This cartilage is unaffected by major pathologies of the diarthrodial joints, and is frequently isolated and utilized in reconstructive surgeries [4-6]. As a hyaline cartilage, costal cartilage provides a differentiated, pure, primary cell population, circumventing the need for differentiation cues employed in conjunction with stem cells, and altogether avoiding associated ethical challenges. Obtaining a purified, chondrogenically differentiated cell population from stem cells continues to be a significant challenge. Stem cells have yet to be differentiated *in vitro* in a consistent fashion to produce type II collagen [7]. Importantly, costal chondrocytes may be expanded *in vitro*, while maintaining the ability to generate hyaline cartilaginous matrix [3,8,9]. While costal chondrocytes demonstrate phenotypic alterations in monolayers similar to articular chondrocytes, including decreased type II collagen and glycosaminoglycan expression [8,10], previous work has shown that expansion and three-dimensional redifferentiation culture conditions may be modulated to enhance hyaline cartilaginous matrix production post expansion [3,8,9]. Specifically, third-passage costochondral cells have demonstrated the ability to self-assemble to generate neocartilage rich in type II collagen and glycosaminoglycans (GAGs) with compressive properties within the range of native temporomandibular joint condylar cartilage [3]. However, engineered neocartilage has yet to completely replicate the collagen content and tensile properties of native tissues. Various biochemical, biophysical, and biomechanical exogenous stimuli have been utilized with alternate cell sources to enhance the functional properties of engineered tissues. Combining exogenous stimuli with a clinically relevant cell source, costal chondrocytes, may improve the translational potential of engineered cartilage.

Hydrostatic pressure (HP) enhances collagen synthesis and the resulting tensile properties in articular chondrocytes [11,12], while its effects on matrix synthesis in costal chondrocytes have not yet been investigated. In cartilage engineered with articular chondrocytes, 10 MPa static HP significantly increased the collagen and GAG content, as well as both compressive and tensile properties [13]. Combining HP and transforming growth factor beta-1 (TGF-β1) led to an additive benefit in compressive and tensile moduli and a synergistic benefit in collagen content [13]. The mechanism of action of HP in articular chondrocytes is not fully characterized, but it is known that HP does not deform cartilage. Rather, HP compresses void spaces surrounding membrane-bound ion channels, and alters channel activity and intracellular ion concentrations [11,14-16]. With changes in intracellular ion concentrations affecting gene expression and protein synthesis [17], HP may initiate downstream upregulation of extracellular matrix-specific genes and protein production [18]. HP may offer an additional means of enhancing the functional properties of expanded, redifferentiated costochondral cell neocartilage.

TGF-β has been investigated for its benefits on chondrocyte matrix synthesis in various systems. TGF-β controls an array of cell processes including cell proliferation, differentiation, and developmental fate [19,20]. In articular chondrocytes, TGF-β1 mediates cell survival and matrix synthesis [21]. This factor has been shown to play a key role in maintenance of chondrocyte phenotype, lubricating properties, and chondrocyte response to mechanical loading [22-24]. Exogenous application of TGF-β1 at 10 ng/ml to self-assembled primary articular chondrocytes increased the GAG content and compressive properties [13]; in fibrochondrocytes, it was shown to increase both the collagen and GAG content along with mechanical properties [25]. In primary costochondral cells, 1 ng/ml TGF-β1 increased proline, thymidine, leucine, and sulfate incorporation [26]. However, 1 ng/ml TGF-β1 had no effect on mechanical properties of expanded costochondral cell constructs [27]. TGF-β1 has also been shown to increase superficial zone protein (SZP) in articular chondrocytes [28]. SZP contributes to boundary lubrication and protects the articular surface from cell and protein adhesion [29-31]. A main objective in tissue engineering of articular cartilage remains achieving lubrication [32]. TGF-β1 may be used to enhance articular chondrocyte protein synthesis *in vitro* but its effect in costochondral cells, specifically at a higher dose, requires further examination.

Chondroitinase ABC (C-ABC) is a matrix remodeling enzyme that facilitates maturational growth in cartilage explants and engineered constructs [33,34]. C-ABC selectively degrades chondroitin and dermatan sulfate [35]. While tensile properties of cartilage are largely associated with the collagen network, the swelling pressure imparted by proteoglycans plays an indirect role in tensile integrity. In bovine articular cartilage explants, C-ABC treatment immediately enhanced tensile stiffness and strength. With further culture (13 days) in serum-containing medium, the GAG content was restored, and collagen density and tensile properties increased [34]. In engineered articular chondrocyte constructs, 2 units/ml C-ABC treatment has been shown to increase collagen density and tensile properties with no observed changes in gene expression [33]. C-ABC is a biophysical, matrix-remodeling enzyme that may have the potential to enhance the maturational growth and tensile properties of engineered costochondral cell constructs.

The translational potential of engineered cartilage relies upon developing tissue capable of withstanding *in vivo* loads upon implantation and utilizing a clinically relevant cell source, such as costochondral cells. This work presents the first systematic analysis of the effects of three salient mediators of cartilage formation: the mechanical stimulus HP, the anabolic stimulus TGF-β1, and the catabolic stimulus C-ABC in engineered articular cartilage. In a full-factorial analysis of variance design, this study assessed the effects of HP (10 MPa static, none), TGF-β1 (10 ng/ml, none), and C-ABC (2 units/ml, none) on the neocartilage matrix content, collagen fibril diameter and density, and mechanical properties. We hypothesized that individually TGF-β1, HP, and C-ABC would significantly increase the collagen content and tensile properties; dual C-ABC/TGF-β1, HP/TGF-β1, and HP/C-ABC treatments would increase tensile properties and collagen content in an additive manner, or greater; and full HP/C-ABC/TGF-β1 treatment would lead to a synergistic increase in collagen content and tensile properties.

## Methods

### Cell isolation and expansion

Costal cartilage was obtained from the four caudal asternal ribs (~5 cm) of Yorkshire–Hampshire cross pigs, *Sus scrofa* (6 months of age) within 24 hours of sacrifice (UC Davis Meat Sciences Facility, Davis, CA, USA). The perichondrium was excised and cartilage was minced in Dulbecco’s modified Eagle’s medium. Tissue was digested in 0.2% collagenase type II (Worthington, Lakewood, NJ, USA) with 3% fetal bovine serum (Atlanta Biologicals, Lawrenceville, GA, USA) for 18 hours at 37°C in chemically defined chondrogenic culture medium (CHG) composed of Dulbecco’s modified Eagle’s medium with 4.5 g/l glucose and GlutaMAX (Gibco, Grand Island, NY, USA), 1% penicillin–streptomycin–fungizone (BD Biosciences, Bedford, MA, USA), 1% ITS+ premix (BD Biosciences), 1% non-essential amino acids (Gibco), 100 nM dexamethasone, 50 μg/ml ascorbate-2-phosphaste, 40 μg/ml l-proline, and 100 μg/ml sodium pyruvate [36]. Cells were filtered (70 μm filter; BD Biosciences), counted, and stored frozen in liquid nitrogen until use; isolations yielded 15 × 10^6^ to 20 × 10^6^ cells/set of four ribs with greater than 90% viability. Cells were thawed and pooled from four animals (73% viability) and seeded in T-225 flasks at 2.5 × 10^4^ cells/cm^2^. Cells were expanded in CHG supplemented with 1 ng/ml TGF-β1 (Peprotech, Rocky Hills, NJ, USA), 10 ng/ml platelet-derived growth factor (Peprotech), and 5 ng/ml basic fibroblastic growth factor (Peprotech). This expansion cocktail was selected based on previous work in chondrocytes demonstrating enhanced proliferation throughout expansion and improved post-expansion chondrogenesis [3,8,37,38]. Cells were passaged at 80 to 90% confluence with 0.5% Trypsin–ethylenediamine tetraacetic acid (Gibco), followed by 0.2% collagenase solution three times (passage 3).

### Redifferentiation and self-assembly

After the third passage, cells were redifferentiated in aggregate culture for 10 days to further enhance post-expansion chondrogenesis. The aggregate redifferentiation technique was selected based on previously demonstrated benefits in articular chondrocytes and meniscus cells [39]. During aggregate culture, cells were maintained on agarose-coated (1% agarose in phosphate-buffered saline; Fisher Scientific, Pittsburgh, PA, USA) plates at 750,000 cells/ml in CHG containing 10 ng/ml TGF-β1 on an orbital shaker for the first 24 hrs. After 10 days, aggregates were digested for 45 minutes in 0.5% Trypsin–ethylenediamine tetraacetic acid, followed by 1 hour in 0.2% collagenase type II solution (as described above) to obtain a single-cell suspension.

Constructs were self-assembled in agarose wells of 5 mm diameter. The self-assembling process was utilized to parallel chondrocyte condensation and development [40], and to circumvent negative effects associated with scaffold-based approaches [2]. 2 × 10^6^ cells were seeded into each well on day 0, and medium was changed daily. At no time were cells embedded within the agarose. After 7 days, constructs were unconfined and moved into wells coated with 2% agarose to prevent adhesion, and media were changed every other day.

### Exogenous stimuli application

Constructs were randomly assigned to each treatment or control group (*n* = 8/group). This study employed a full factorial 3 × 2 design: C-ABC (2 units/ml, no C-ABC); TGF-β1 (10 ng/ml, no TGF-β1); and HP (10 MPa static, no HP) (see Table 1). Groups receiving C-ABC were treated with 2 units/ml C-ABC in CHG (4 ml per eight constructs) for 4 hours on day 15. C-ABC was activated with 0.05 M sodium acetate (Sigma, St. Louis, MO, USA) and inactivated with 1 mM Zn^2+^ (Sigma). Constructs receiving TGF-β1 were treated continuously throughout culture at 10 ng/ml.

**Table 1:** Exogenous treatment

**Treatment**	**Control**	**HP**	**C-ABC**	**TGF-β1**	**HP/C-ABC**	**HP/TGF-β1**	**TGF-β1/C-ABC**	**HP/C-ABC/TGF-β1**
HP: 10 MPa, static, days 10 to 14		x			x	x		x
C-ABC: 2 units/ml, 4 hours, day 15			x		x		x	x
TGF-β1: 10 ng/ml, continuous, days 0 to 28				x		x	x	x

C-ABC, chondroitinase ABC; HP, hydrostatic pressure; TGF-β1, transforming growth factor beta-1.

For the application of HP, a custom bioreactor was assembled as described previously [41]. Briefly, HP treatment consisted of heat-sealing constructs in sterilized bags (Kapak, Minneapolis, MN, USA) containing CHG (with or without TGF-β1). Sealed bags were submerged in a 1 L stainless steel pressure vessel and pressurized to 10 MPa for 1 hour at 37°C for 5 consecutive days (days 10 to 14 of culture) [11]. After treatment, constructs were returned to normal culture conditions.

### Histology and biochemistry

Construct samples were evaluated after 4 weeks of culture. Samples from each treatment group, as well as mature porcine articular and costal cartilage, were frozen in Histoprep Frozen Tissue Embedding Media (Fisher Scientific). Samples were sectioned at 14 μm and stained with picrosirius red for collagen or Safranin-O/fast-green for GAGs. Additionally, samples were assessed immunohistologically for type I and type II collagen, as described previously [3]. Samples were assessed for SZP using mouse anti-PRG4 monoclonal antibody (clone 2A6) at 1:100 dilution (Sigma).

Collagen, GAG, and DNA contents were quantified in engineered cartilage. Samples were digested in 125 μg/ml papain (Sigma) in phosphate buffer (pH 6.5). Samples were hydrolyzed in 4 N NaOH for 20 minutes at 110°C, and a modified hydroxyproline assay was used to quantify the collagen content. A Blyscan glycosaminoglycan assay kit (Biocolor, Westbury, NY, USA) was used to quantify sulfated GAG, and cellularity was quantified using the Quant-iT Picrogreen double-stranded DNA assay kit (Invitrogen, Carlsbad, CA, USA).

### Collagen fibril analysis

Samples from each group and from native porcine costal cartilage and articular cartilage were fixed in 3% glutaraldehyde (Sigma) in cacodylate buffer and stored at 4°C. Immediately prior to imaging, specimens were dehydrated in ascending exchanges of ethanol (25%, 50%, 75%, 100%). Samples were critical point dried, mounted, sputter coated, and imaged with a Philips XL30 TMP (F.E.I. Company, Hillsboro, OR, USA) scanning electron microscope (SEM). After imaging, ImageJ (National Institute of Health, Bethesda, MD, USA) analysis software was used to measure the fibril density and diameter. The threshold function was used to set threshold limits and the measure function was used to quantify the percentage area occupied by fibrils, which is reported as the fibril density. Also using the threshold and measure functions, 10 fibrils were randomly selected, and their diameters were measured in each of six images per group.

### Mechanical evaluation

Mechanical properties were evaluated in tension and compression. Compression samples consisted of 2 mm punches from the central region of each construct. Additionally, 2 mm diameter compression samples were taken from porcine costal cartilage and articular cartilage (samples obtained from four animals). In compressive testing, samples were preconditioned with 15 cycles of 5% compressive strain and then strained to 10% and 20% deformation, sequentially in a stress-relaxation test using an Instron 5565 (Instron, Norwood, MA, USA). A Kelvin solid viscoelastic model was fit to the data to establish compressive material properties at each strain level as described previously [42]. Values for the instantaneous modulus, relaxation modulus, and coefficient of viscosity were quantified. Tensile testing consisted of a uniaxial pull-apart test and was conducted using a Test Resources 840 L (Test Resources, Shakopee, MN, USA). A dogbone-shaped specimen was obtained by taking a second 2 mm punch adjacent to the first. This procedure was repeated with costal cartilage and articular cartilage to obtain native tissue values (samples obtained from four animals). Paper tabs were used to establish a consistent gauge length of 1.4 mm. Samples were elongated at a strain rate of 1%/s. Stress–strain curves were developed from the load–displacement curve. Young’s modulus and the ultimate tensile strength (UTS) were quantified.

### Statistical analysis

Three-way analysis of variance was used to identify the presence of significant differences in biochemical and biomechanical properties (α = 0.05). Where indicated, Tukey’s *post-hoc* test was used to identify differences between specific treatment groups (*P* <0.05). JMP 10 software (SAS Institute Inc., Cary, NC, USA) was used to carry out statistical analysis. Groups reported not connected by lowercase letters are significantly different. All data are reported as mean ± standard deviation. Synergism is reported as a combined treatment effect greater than the sum of the independent effects of each stimulus – that is:

$$\left(\mu_{\mathrm{AB}}–\mu_{\mathrm{CONTROL}}\right)>\left[\left(\mu_{A}–\mu_{\mathrm{CONTROL}}\right)+\left(\mu_{B}–\mu_{\mathrm{CONTROL}}\right)\right])$$

where μ is the average for each group. The presence of statistically significant correlations was determined using Pearson’s correlation coefficient with a two-tailed probability level (*P* <0.05).

## Results

### Gross morphology and histology

**Figure 1:**
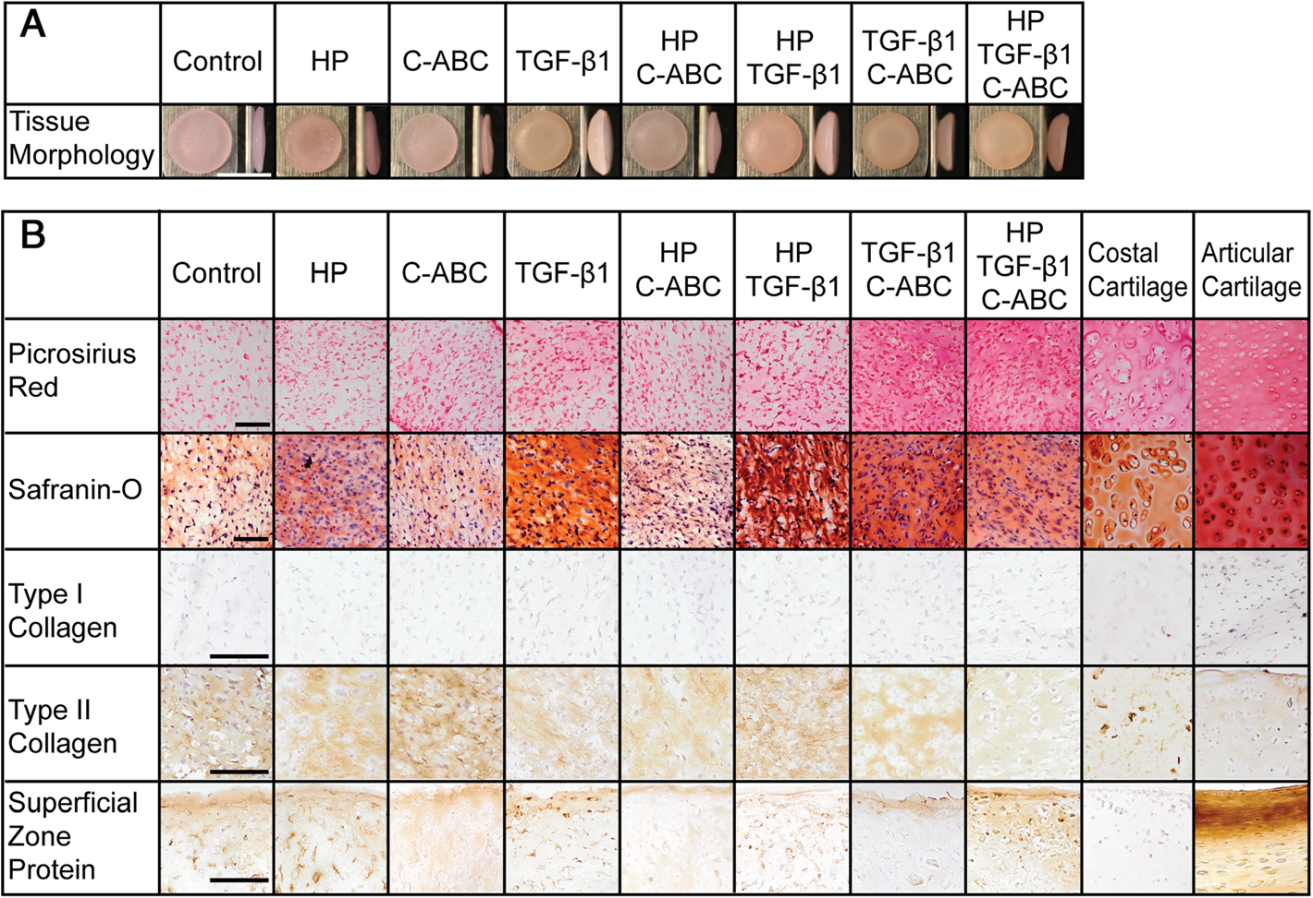
**Neocartilage morphology and histology. (A)** A flat disc-shaped construct was observed in control, hydrostatic pressure (HP) and chondroitinase ABC (C-ABC) treatments. The addition of transforming growth factor beta-1 (TGF-β1) resulted in a bowl-shaped construct. **(B)** Histological staining demonstrated the presence of collagen (picrosirius red) and glycosaminoglycans (Safranin-O/fast-green) in all groups. Immunohistochemistry demonstrated the presence of type II collagen, and the absence of type I collagen, independent of the treatment regimen. Immunohistochemical staining for superficial zone protein (SZP) demonstrated its presence in the superficial zone of articular cartilage, and its absence in costal cartilage. Engineered neocartilage demonstrated the presence of SZP independent of treatment regimen. Morphology scale bar = 5 mm, histology scale bar = 100 μm.

Gross morphology and histological staining are shown in Figure 1. Homogeneous cylindrical cartilaginous tissues were generated in all treatments. TGF-β1 and combinations containing TGF-β1 yielded a bowl-shaped morphology that was associated with decreased construct diameter. Control constructs demonstrated the largest tissue diameter (5.9 ± 0.1) (Table 2). Histology and immunohistochemistry showed an extracellular matrix rich in GAG and collagen, specifically positive for type II collagen and negative for type I collagen (Figure 1B). In control and HP constructs, collagen staining was primarily pericellular. In combinatorial treatments C-ABC/TGF-β1 and HP/C-ABC/TGF-β1, collagen staining was more intense and homogeneously distributed. With C-ABC treatment, the GAG content was similar to control, but the GAG content appeared greater than control in C-ABC/TGF-β1 and HP/C-ABC/TGF-β1 treatments. Shown in Figure 1B, immunohistochemistry confirmed the presence of SZP in the superficial zone of porcine articular cartilage and demonstrated its absence in costal cartilage. Independent of the treatment regimen, neocartilage stained positively for SZP.

**Table 2:** Neocartilage dimensions, hydration, and cellularity

**Treatment**	**Control**	**HP**	**C-ABC**	**TGF-β1**	**HP/C-ABC**	**HP/TGF-β1**	**TGF-β1/C-ABC**	**HP/C-ABC TGF-β1**
Diameter (mm)	5.9 ± 0.1^a^	5.8 ± 0.1^a^	5.5 ± 0.1^b^	5.6 ± 0.0^b^	5.6 ± 0.1^b^	5.6 ± 0.0^b^	5.3 ± 0.1^c^	5.4 ± 0.1^c^
Thickness (mm)	0.7 ± 0.1^ab^	0.7 ± 0.1^a^	0.5 ± 0.1^d^	0.7 ± 0.1^abc^	0.5 ± 0.0^d^	0.7 ± 0.0^a^	0.5 ± 0.0^cd^	0.6 ± 0.0^bcd^
Hydration (% water)	89.2 ± 0.6^a^	89.4 ± 0.9^a^	86.4 ± 0.9^b^	86.3 ± 0.6^b^	86.2 ± 0.5^b^	86.8 ± 0.6^b^	86.8 ± 0.7^b^	86.1 ± 1.4^b^
Cells × 10^6^/construct	1.6 ± 0.4^d^	1.8 ± 0.2^cd^	2.0 ± 0.1^abcd^	2.0 ± 0.3^bcd^	2.1 ± 0.2^abc^	2.2 ± 0.3^abc^	2.4 ± 0.3^ab^	2.4 ± 0.3^a^

Data reported as mean ± standard deviation. C-ABC, chondroitinase ABC; HP, hydrostatic pressure; TGF-β1, transforming growth factor beta-1. Lowercase letters denote significance. All groups not connected by a common letter are significantly different (*P* <0.05).

### Biochemical content

**Figure 2:**
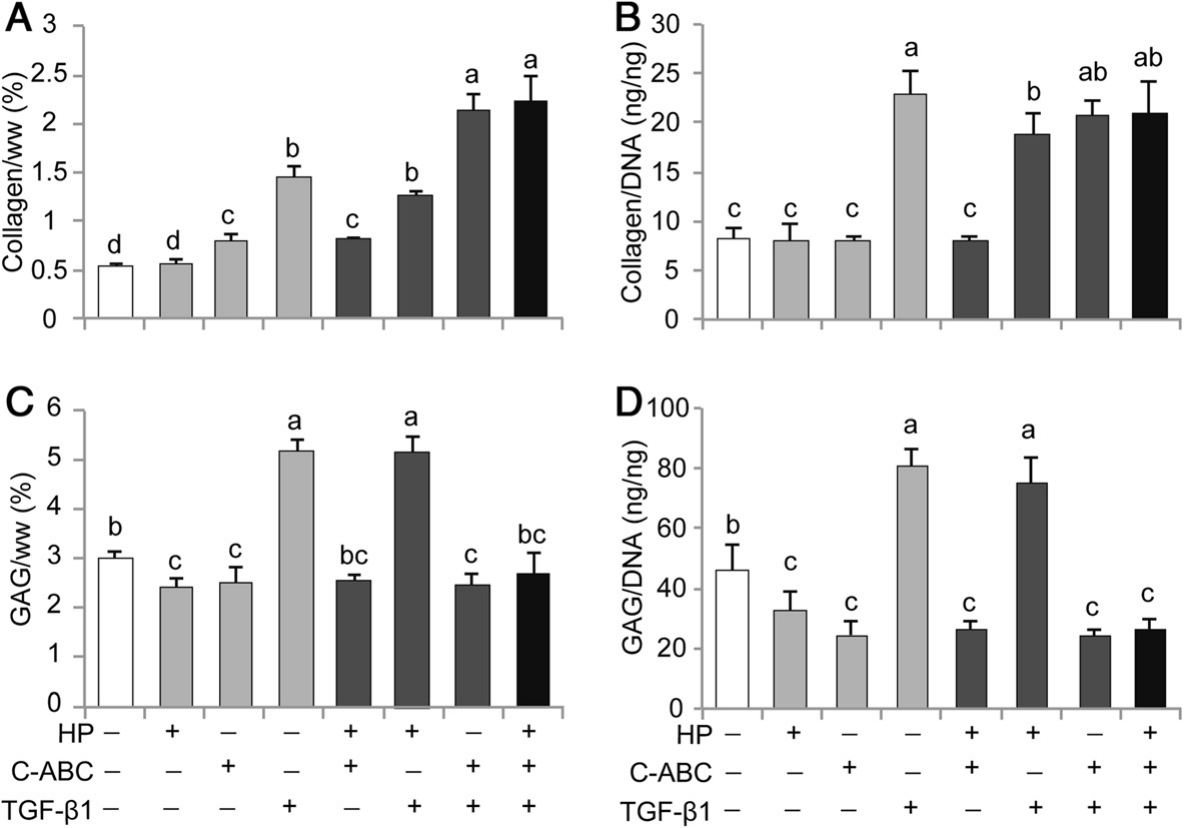
**Biochemical composition of neocartilage normalized to tissue wet weight and DNA. (A)** Chondroitinase ABC (C-ABC) and transforming growth factor beta-1 (TGF-β1) individually enhanced collagen density (per wet weight (ww)), and C-ABC/TGF-β1 and hydrostatic pressure (HP)/C-ABC/TGF-β1 treatments synergistically enhanced collagen density (per ww). **(B)** Reflecting phenotypic changes, TGF-β1 led to the greatest increase in collagen production per cell. **(C)**, **(D)** TGF-β1 additionally enhanced the glycosaminoglycan (GAG) content, normalized to ww and DNA, while C-ABC and combined treatments containing C-ABC showed the lowest GAG/DNA. Data reported as mean ± standard deviation. All groups not connected by a common lowercase letter are significantly different (*P* <0.05).

Collagen, GAG, DNA, and water contents are reflected in Figure 2 and Table 2. The collagen content significantly increased with all single treatments, only trending higher with HP. Collagen content was greatest in the presence of C-ABC/TGF-β1 (2.1 ± 0.2%) and HP/C-ABC/TGF-β1 (2.2 ± 0.3%) treatments. As factors, only TGF-β1 significantly increased collagen/DNA while both C-ABC and TGF-β1 significantly increased collagen/wet weight. Compared with control, the GAG content was reduced with C-ABC (2.5 ± 0.3%) and HP (2.4 ± 0.2%), while it was significantly increased with TGF-β1 (5.2 ± 0.2%) and HP/TGF-β1 treatment (5.2 ± 0.3%). The HP/C-ABC and HP/C-ABC/TGF-β1 treatments recovered the GAG content to control values. C-ABC and TGF-β1 were significant factors in GAG/wet weight, with TGF-β1 increasing GAG and C-ABC decreasing GAG. Cellularity trended higher than control with all single and combined treatments, and was greatest in HP/C-ABC/TGF-β1 treatment (2.4 ± 0.3 × 10^6^ cells/construct). Water content was greatest in control and HP-treated constructs (Table 2). Hydration was significantly reduced with C-ABC and TGF-β1 alone, and with all combinatorial treatments.

### Collagen fibril density and diameter

**Figure 3:**
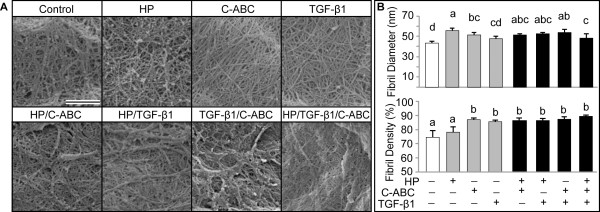
**Scanning electron microscopy images and fibril analysis in neocartilage. (A)** Samples were fixed and imaged after 4 weeks culture. Scale bar = 1 μm. **(B)** Chondroitinase ABC (C-ABC), hydrostatic pressure (HP), and all combinatorial treatments significantly increased the fibril diameter. Fibril density was significantly increased over control in all treatments with the exception of hydrostatic pressure. Data reported as mean ± standard deviation. All groups not connected by a common lowercase letter are significantly different (*P* <0.05). TGF-β1, transforming growth factor beta-1.

Collagen was imaged via SEM (Figure 3A) and the fibril diameter and density were quantified (Figure 3B). The fibril diameter significantly increased with HP (55.8 ± 2.0 nm) and C-ABC (51.1 ± 2.9 nm) alone, and with all combinatorial stimuli, compared with control. Fibril density increased significantly with C-ABC (87.1 ± 1.1%) and TGF-β1 (85.6 ± 1.1%) alone, and with all combinatorial treatments. HP, C-ABC, and TGF-β1 as factors significantly increased fibril density, while HP as a factor significantly increased fibril diameter. Native porcine costal cartilage demonstrated an average fibril diameter of 69.3 ± 3.8 nm while articular cartilage demonstrated a fibril diameter of 61.2 ± 4.8 nm. Fibril density was found to be 88.6 ± 1.4% in costal cartilage and 86.2 ± 0.8% in articular cartilage.

### Mechanical properties

**Figure 4:**
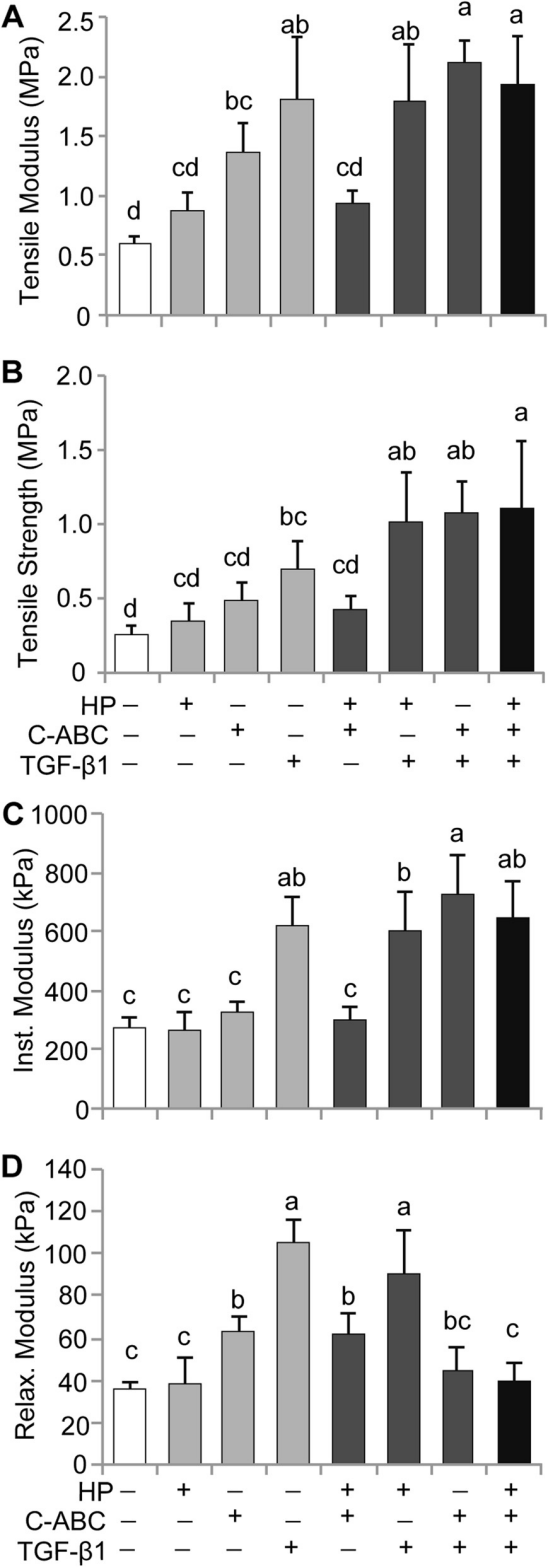
**Tensile and compressive mechanical properties of neocartilage. (A)**, **(B)** Chondroitinase ABC (C-ABC)and transforming growth factor beta-1 (TGF-β1) significantly increased tensile stiffness and strength, while tensile properties trended higher with hydrostatic pressure (HP). The C-ABC/TGF-β1 and HP/C-ABC/TGF-β1 treatments synergistically enhanced tensile strength. **(C)**, **(D)** TGF-β1 also significantly increased compressive instantaneous and relaxation moduli. Combined C-ABC/TGF-β1 treatment synergistically increased instantaneous modulus. GAG depletion with C-ABC treatment did not compromise compressive properties below control. Data reported as mean ± standard deviation. All groups not connected by a common lowercase letter are significantly different (*P* <0.05).

Tensile and compressive properties of engineered tissues are shown in Figure 4. Tensile stiffness increased significantly with C-ABC and TGF-β1 treatments alone, and trended higher with HP. Greatest stiffness was seen in C-ABC/TGF-β1 (2.1 ± 0.2 MPa) and HP/C-ABC/TGF-β1 (1.9 ± 0.4 MPa) treatments; in both treatments, the combinatorial stimuli exceeded the effects of any single stimulus. The UTS trended higher with HP and C-ABC treatments alone, and significantly increased with TGF-β1. The HP/TGF-β1, C-ABC/TGF-β1, and HP/C-ABC/TGF-β1 treatments synergistically increased the UTS; each combination increased the UTS in excess of the cumulative effect of the single treatments. The instantaneous compressive modulus significantly increased in the presence of TGF-β1 (615 ± 105 kPa) and synergistically increased in TGF-β1/C-ABC treatment (727 ± 134 kPa). The relaxation modulus significantly increased with C-ABC and TGF-β1. HP/TGF-β1 was the combination treatment with the greatest relaxation modulus (91 ± 21 kPa). As factors, C-ABC and TGF-β1 significantly increased tensile moduli and strength, and compressive instantaneous moduli. Additionally, statistically significant positive correlations between collagen content per tissue wet weight and tensile stiffness (*R*^2^ = 0.59, *P* <0.001) and between collagen content per tissue wet weight and strength (*R*^2^ = 0.57, *P* <0.001) were detected in engineered neocartilage.

Mature porcine articular and costal cartilage were tested in tension and demonstrated tensile moduli of 22.0 ± 3.9 MPa and 6.4 ± 2.1 MPa, and tensile strengths of 11.0 ± 1.8 MPa and 2.9 ± 0.9 MPa, respectively. In compression, porcine articular and costal cartilage demonstrated relaxation moduli of 190 ± 50 kPa and 720 ± 390 kPa, and instantaneous moduli of 5.4 ± 0.5 MPa and 3.3 ± 1.6 MPa, respectively.

## Discussion

This study sought to investigate additive and synergistic benefits of combined anabolic and catabolic stimuli toward enhancing the functional properties of neocartilage engineered using clinically relevant costochondral cells. Costal cartilage offers a useful donor cell population that is unaffected by diseases of diarthrodial joints. Costal cartilage is currently isolated with minimal donor site morbidity for use in reconstructive surgeries, and improvements in the biomechanical properties of cartilage engineered with costochondral cells may allow for use in load-bearing joints. Toward this, the independent effects of TGF-β1, C-ABC, and HP and their combinatorial benefits were examined in third-passage, redifferentiated costochondral cell constructs. The overall hypothesis was that expanded, redifferentiated costochondral cells would respond beneficially to exogenous stimuli by demonstrating enhanced collagen content and tensile properties. The results of this study confirmed the hypothesis, showing that TGF-β1 and C-ABC independently enhanced collagen content and tensile properties of engineered constructs. Also, dual treatments further enhanced properties over single treatments. Furthermore, the effects of the full HP/C-ABC/TGF-β1 treatment were more pronounced than dual treatments, except for C-ABC/TGF-β1. Costochondral cells present a clinically relevant cell source that, when expanded, redifferentiated, and self-assembled, respond to exogenous stimuli to generate mechanically robust tissue suitable for load-bearing joints.

TGF-β1 treatment significantly increased the collagen and GAG contents and both tensile and compressive mechanical properties of expanded, redifferentiated costochondral cell constructs. Previously, low-dose TGF-β1 stimulation (1 ng/ml) of primary costochondral cells increased proline, thymidine, leucine, and sulfate incorporation [26]. However, in expanded, costochondral cells, low-dose TGF-β1 had no effect on mechanical properties of engineered tissue [27]; this dose was an order of magnitude lower than that used here. Additionally, the costochondral cells in the present study underwent aggregate redifferentiation following expansion, resulting in the production of type II collagen, GAG, and SZP akin to articular chondrocytes (Figure 1). In articular chondrocytes, TGF-β1 signaling has been shown to be dose dependent, with concentrations greater than 1 ng/ml increasing type II collagen, aggrecan, and SZP secretion [28,43]. In the present study, TGF-β1 stimulation at 10 ng/ml significantly increased biochemical content and mechanical properties of engineered costochondral cell tissue.

C-ABC enhanced collagen density, fibril diameter, and tensile properties in engineered costochondral cell neocartilage. While C-ABC did not affect collagen synthesis per cell (Figure 2B), the total collagen content per tissue wet weight increased by 50%. SEM analysis of the matrix revealed that C-ABC significantly increased fibril diameter by 18% and density by 17%. With C-ABC treatment, collagen fibrils on average were 51.1 ± 3.0 nm, approaching that of mature porcine articular cartilage (61.2 ± 4.8 nm). Additionally, increased fibril diameter has previously been shown to correlate positively with tensile modulus [44]. This supports the hypothesis that the 125% increase in tensile modulus with C-ABC treatment resulted from biophysical changes including increased fibril diameter and density.

C-ABC is suggested to act on a biophysical level through the temporary depletion of small proteoglycans to enhance tensile properties. In articular chondrocytes, C-ABC similarly increased the fibril diameter and density, while no effect on genetic signaling was observed [33,45]. Small collagen-binding proteoglycans, whose GAG chains are cleaved by C-ABC, are known to play a role in collagen fibrillogenesis [46,47]. One such proteoglycan, decorin, mediates the fibril diameter and the interaction between fibrils [47], including fibril adhesion and sliding. In the present study, GAG depletion may allow changes in the matrix organization as well as fibrillogenesis, as evidenced by the compact, aligned matrix seen with C-ABC treatment and the increased fibril diameter. In self-assembled costochondral cells, C-ABC is suggested to act through the temporary depletion of proteoglycans to alter matrix characteristics and enhance tensile properties.

TGF-β1/C-ABC dual treatment synergistically enhanced the collagen content and tensile strength in expanded costochondral cell constructs. The combination of C-ABC and TGF-β1 increased collagen density per wet weight by 300% over control, which was notably greater than the effect of TGF-β1 (170%) or C-ABC (50%) alone. As a result of the observed matrix changes, the combined stimuli enhanced tensile stiffness by 250% and strength by 320%, over control. In articular chondrocytes, TGF-β1 has been shown to act in the canonical pathway via SMAD signaling to upregulate type II collagen synthesis [48,49], while C-ABC has been shown to act on a nongenetic level [33] to increase fibril density and diameter. In costochondral cell constructs, the combination of an anabolic agent that enhances biosynthesis (TGF-β1) and a catabolic agent that acts in a biophysical manner to increase fibril density (C-ABC) synergistically enhanced collagen content and tensile strength.

HP increased the collagen fibril diameter and density in costochondral cell constructs. Analysis of SEM images revealed that HP increased the fibril diameter by 30%; this was the greatest increase in fibril diameter observed with any treatment. HP also significantly increased the fibril density. In articular chondrocytes, HP has previously been shown to increase the collagen content and tensile properties [13,18], while the fibril diameter and density were not investigated. In the present system, HP as a factor did not significantly increase tensile properties, although a trending increase in tensile strength was observed (*P* = 0.12). Additional investigation is required to identify whether HP has a significant effect in this cell system and whether alternate loading conditions produce more beneficial effects. Mechanisms downstream of ion channel-based alterations may be one means by which HP increases fibril diameter and density in costochondral cell constructs.

The extracellular signal-regulated kinase 1/2 (ERK1/2) pathway may be a second mechanism of action for both HP and TGF-β1, with TGF-β1 responding more robustly. In treatments containing both HP and TGF-β1, the biomechanical benefits of HP were dominated by TGF-β1. Previous work with articular chondrocytes stimulated by HP via the regimen used here demonstrated that the ERK1/2 pathway is required for tensile property enhancement [18]. Inhibition of ERK1/2 by U0126 blocked the tensile modulus enhancement observed with HP stimulation. TGF-β1 has also been shown to activate matrix production in articular chondrocytes via ERK1/2 [50]. In the combined HP/TGF-β1 treatment, the collagen and GAG contents and mechanical properties showed no significant differences from TGF-β1 treatment alone. Additionally, no significant differences were observed between C-ABC/TGF-β1 and full HP/C-ABC/TGF-β1 treatment in biochemical content or mechanical properties. With both of these stimuli showing action through the ERK1/2 pathway in articular chondrocytes, the effect of TGF-β1 may be more robust in this cell population.

Engineered costochondral cell neocartilage demonstrated tensile properties that correlated with collagen content. In the present study, biomechanical, biophysical, and biochemical stimuli were employed with an objective of engineering robust tissues that would be capable of withstanding *in vivo* loads from cells that normally do not bear such loads. The results demonstrated that TGF-β1 upregulated collagen synthesis (175% increase in collagen/DNA) associated with increased tensile properties. In contrast, C-ABC led to no change in collagen synthesis on the cell level, yet increased tensile properties through modulation of fibril diameter and density. The statistically significant positive correlation between collagen content per tissue wet weight and tensile stiffness (*R*^2^ = 0.59, *P* <0.001) and strength (*R*^2^ = 0.57, *P* <0.001) is thus a function of both collagen synthesis and fibril compaction. Full HP/C-ABC/TGF-β1 treatment achieved 2.2% collagen/wet weight and a tensile modulus of 2 MPa. One may anticipate that further efforts to enhance collagen production, maturation, and organization will result in further increases in tensile properties of engineered tissues.

Costochondral cells present a clinically relevant cell source that may be stimulated *in vitro* to generate robust articular cartilage for use in load-bearing joints. Costal cartilage may be isolated with ease surgically, and is unaffected by pathologies of the articulating joints, including arthritis. Costochondral cells may be expanded in monolayer to increase cell number, and, furthermore, chondrogenic redifferentiation and self-assembly result in a cell population that produces markers of articular cartilage: type II collagen, GAG, and SZP. While SZP gene [51] and protein (Figure 1) expression is absent in costal cartilage natively, engineered neocartilage demonstrated the presence of this protein, which functions in lubrication in load-bearing, diarthrodial joints. Additionally, expanded, redifferentiated costal chondrocytes respond to exogenous stimuli similarly to articular chondrocytes [13,28,33,34]. Most notably, costal chondrocytes show a beneficial response to TGF-β1, C-ABC, and HP individual treatments, and a synergistic increase in tensile strength and collagen content in dual C-ABC/TGF-β1 treatment. The presence of SZP in engineered neocartilage further suggests that nonarticular costochondral cells may be induced to act in a manner reminiscent of articular chondrocytes. Expanded, redifferentiated costochondral cells respond beneficially to exogenous stimuli to generate robust articular cartilage, indicating the potential of this cell source in engineering load-bearing joint structures.

## Conclusions

This study presents the first systematic analysis of the independent and combinatorial benefits of salient biochemical, biomechanical, and biophysical stimuli in engineering costochondral cell neocartilage tissue replacements. Moreover, this analysis was performed using a clinically relevant cell population, costochondral cells, which are unaffected by pathologies of articulating joints. HP, TGF-β1, and C-ABC each enhanced functional properties of engineered tissues, and dual treatments further enhanced the collagen content, and tensile and compressive properties. Overall, full HP/C-ABC/TGF-β1 treatment achieved a tensile modulus of 2 MPa, an instantaneous compressive modulus of 650 kPa, and a relaxed modulus of 40 kPa with a matrix composition most similar to native articular cartilage.

## Abbreviations

C-ABC: Chondroitinase ABC; CHG: Chondrogenic culture medium; ERK1/2: Extracellular signal-regulated kinase 1/2; GAG: Glycosaminoglycan; HP: Hydrostatic pressure; SEM: Scanning electron microscope; SZP: Superficial zone protein; TGF-β1: Transforming growth factor beta-1; UTS: Ultimate tensile strength.

## Competing interests

The authors declare that they have no competing interests.

## Authors’ contributions

MKM, GDD, JCH, and KAA were responsible for conception of design. MKM performed all cell culture, and biochemical and mechanical assays, and scanning electron imaging. MKM and GDD performed histological staining. MKM and GKD drafted the manuscript. AHR, JCH, and KAA critically revised the manuscript. All authors contributed to conception of the design, data acquisition, or analysis; analyzed and interpreted data; contributed to manuscript drafting and critical revision; and read and approved the final manuscript.
